# Windup of Nociceptive Flexion Reflex Depends on Synaptic and Intrinsic Properties of Dorsal Horn Neurons in Adult Rats

**DOI:** 10.3390/ijms20246146

**Published:** 2019-12-05

**Authors:** Franck Aby, Rabia Bouali-Benazzouz, Marc Landry, Pascal Fossat

**Affiliations:** Department Neurocampus, Institut Interdisciplinaire des Neurosciences, Université de Bordeaux, IINS CNRS UMR 5297, 146 rue Léo Saignat, CEDEX, 33077 Bordeaux, France; koffi-franck.aby@u-bordeaux.fr (F.A.); rabia.benazzouz@u-bordeaux.fr (R.B.-B.); marc.landry@u-bordeaux.fr (M.L.)

**Keywords:** pain, central sensitization, L-type calcium channels, NMDA receptors, glycine, dorsal horn of the spinal cord

## Abstract

Windup, a progressive increase in spinal response to repetitive stimulations of nociceptive peripheral fibers, is a useful model to study central sensitization to pain. Windup is expressed by neurons in both the dorsal and ventral horn of the spinal cord. In juvenile rats, it has been demonstrated both in vivo and in vitro that windup depends on calcium-dependent intrinsic properties and their modulation by synaptic components. However, the involvement of these two components in the adults remains controversial. In the present study, by means of electromyographic and extracellular recordings, we show that windup in adults, in vivo, depends on a synaptic balance between excitatory N-methyl-D-aspartate (NMDA) receptors and inhibitory glycinergic receptors. We also demonstrate the involvement of L-type calcium channels in both the dorsal and ventral horn of the spinal cord. These results indicate that windup in adults is similar to juvenile rats and that windup properties are the same regardless of the spinal network, i.e., sensory or motor.

## 1. Introduction

The dorsal horn of the spinal cord is the first relay of nociceptive inputs rising from the periphery. Dorsal horn neuronal networks integrate nociceptive information, and output neurons project both to supraspinal areas and motoneurons. Within this network, deep dorsal horn neurons (DHNs) receive convergent inputs from tactile and nociceptive fibers and express a form of short-term activity-dependent plasticity, so-called windup. Windup is a form of early onset spinal sensitization [1,2] and is considered to be a simple tool to study pain-processing plasticity in the spinal cord (for reviews, see [3,4]. In clinics, windup protocol serves to evaluate temporal summation of pain and is considered to be a good predictor for clinical pain intensity in idiopathic pain syndromes, such as fibromyalgia [5,6]. Windup is also expressed in motor network and repetitive nociceptive stimulations of the hind-paw elicit windup of segmental reflexes [7]. Moreover, repetitive stimulations of the sciatic nerve elicit windup of ventral roots [4]. Finally, motor neurons express windup in different animal models, such as rats or turtles [8,9].

At the level of the dorsal horn, windup is expressed by wide dynamic range neurons and has been extensively studied. In adult rats, it has been shown that windup depends on excitatory synaptic properties as NMDA glutamate receptors and neurokinin receptors. [10]. Windup also depends on DHNs intrinsic properties since windup is blocked by l-type calcium channels (LTCs) blockers and increased by activators of these channels [11]. Other studies performed in spinal slices from juvenile rats confirmed the role of LTCs in this phenomenon and also show the involvement of another cationic nonspecific conductance (CAN) [12]. DHNs project to the ventral horn network, activating motor neurons eliciting muscular contraction necessary for nociceptive flexion reflex [7]. Repetitive nociceptive stimulations of the ipsilateral paw elicit a windup of nociceptive flexion. It has been shown that windup of the flexion reflex in juvenile rats depends on a synaptic balance between excitation and inhibition that allows expression of intrinsic properties [11,13,14]. However, the flexion reflex is the output of a neural circuit that comprises both DHNs and motor neurons, and the latter ones also exhibit a windup of their discharge that presents the same molecular sensitivity as windup of DHNs [8,9,15]. Because of the different model used (juvenile versus adult, turtles), the site of recordings (DHNs, motor neurons, and flexion reflex), our understanding of windup remains partial. For instance, it is still unknown if the balance between excitation and inhibition also control windup at the level of the dorsal horn of the spinal cord. The involvement of intrinsic properties in the windup of the flexion reflex in adult rats is still controversial [4]. Finally, previous studies on reflex have been performed in juvenile rats, and we cannot exclude developmental modifications altering windup molecular sensitivity.

We propose, in this study, to analyze the windup for DHNs in anesthetized rats, by means of extracellular recordings and the windup of flexion reflex with electromyographic recordings in the biceps femoris muscle of the ipsilateral hind paw and compare its molecular sensitivity between adult and juvenile rats.

## 2. Results

### 2.1. Windup of WDR Neurons and of the Flexion Reflex Share Similar Characteristics

**Figure 1:**
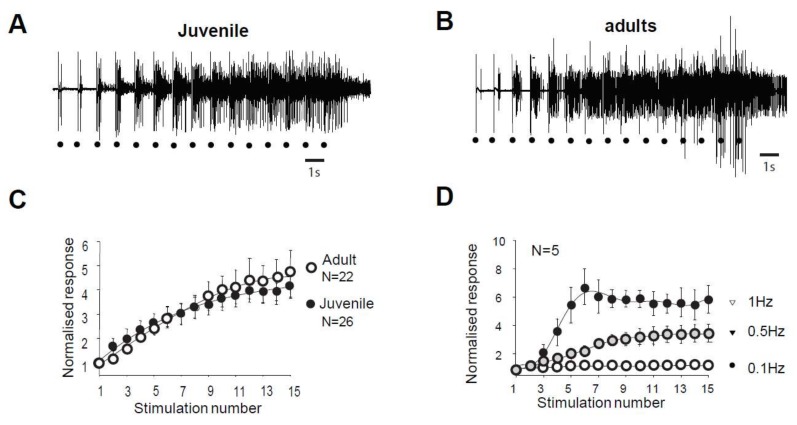
Flexion reflex in adult and juvenile rats (**A**,**B**) Electromyographic recordings (EMG) of windup triggered by a series of electrical shocks in the sural nerve peripheral receptive field in juvenile (**A**) and in adult rats (**B**). Each dot indicates an electric stimulation (500 μs, 80% of the maximal response). (**C**) Normalized windup curves in juvenile (open dots) and adult rats (filled dots). Note that the two curves are strictly superimposed, showing a comparable sensitization. (**D**) In adult rats, windup is frequency-dependent, with no windup for frequency <0.1Hz and a progressive increase until at least 1Hz.

In juvenile rats, electrical stimulation of high threshold fibers in the paw triggers a muscular contraction of the flexor ipsilateral muscle. This response, called RIII flexion reflex or nociceptive flexion reflex, appears in the electromyogram with a latency of 80–300 ms with high stimulations (Figure 1A). The same type of reflex is also observed in adult rats (Figure 1B). Repetitive stimulation at low frequency (here 1 Hz) generates a gradual increase in the reflex magnitude during the sequence of stimulation, with a progressive development of an after-discharge (Figure 1A,B). This increased response is so-called windup. The amplitude of the windup is similar in juvenile and adult rats since the last response of the series represents 467 ± 97% in the adult and 453 ± 56% (*N* = 22 and 26, respectively, *p* > 0.05, Mann–Whitney) in juvenile rats. Windup coefficient, defined as the sum of the 15 responses subtracted by 15 times the first response, is also very similar (Figure 1C, windup coefficient is 30.38 ± 5.77 in adult and 32.04 ± 6.99 in juvenile; *p* > 0.05, Mann–Whitney). Windup of the flexion reflex is frequency-dependent in adult rats (Figure 1D). Together, these results show that windup phenomenon is not modified during development and exhibits the same characteristics at the level of the muscle or the dorsal horn of the spinal cord.

### 2.2. Windup of the Nociceptive Flexion Reflex in Adult Rats is Controlled by Synaptic and Intrinsic Components

**Figure 2:**
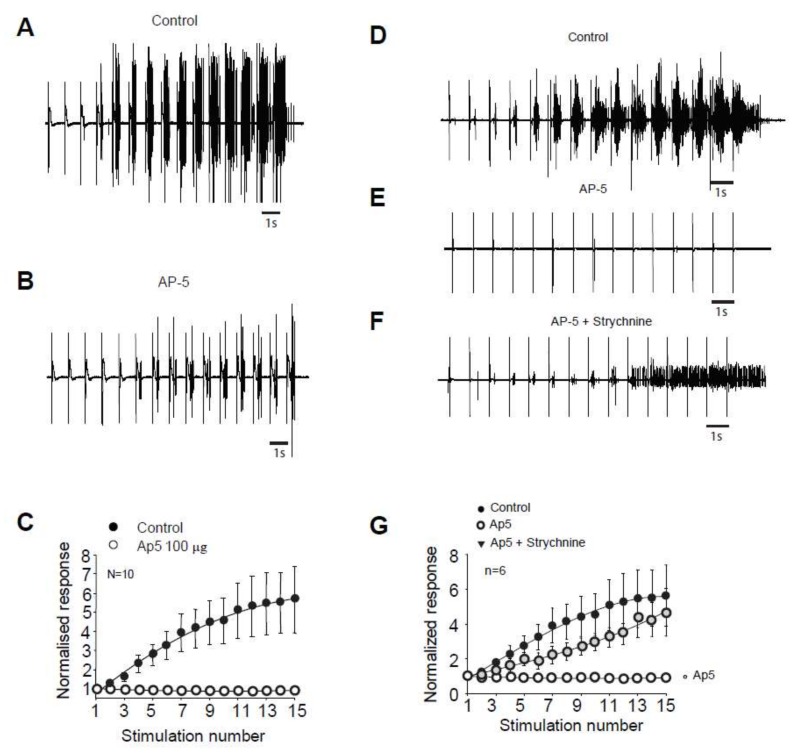
Windup of the nociceptive flexion reflex in adult rats is controlled by NMDAr and glycine receptors. (**A**) EMG in control or (**B**) after application of 100 µg of AP5. (**C**) Windup is significantly decreased by AP5 (windup coefficient: 20.4 ± 4 in control vs. 1.2 ± 0.5 in AP5, *n* = 10, *p* < 0.01, paired t-test). (**D**) EMG in control, (**E**) after 100 µg AP5, and (**F**) after 100 µg AP5 + 170 µg strychnine. (**G**) Application of AP5 suppressed the windup that was restored after subsequent application of AP5 and Strychnine (windup coefficient: 41 ± 13.8 in control vs. –1.39 ± 1 in AP5 and 24.2 ± 7.2 in AP5 + Strychnine, *n* = 6, *p*-control vs. AP5 < 0.01; *p*-control vs. AP5 + strychnine > 0.05, Dunn’s post hoc test).

We next study the molecular mechanisms controlling the windup of the flexion reflex in adult rats. First, we focused on synaptic components, and we observed that an application of 100 µg of AP5 strongly decreased the flexion reflex windup (Figure 2A–C). This effect is dose-dependent with an EC50 of 10.26 µg (not shown). The baseline response was also modified with a significant decrease at 57% of the control. This result is comparable to a previous study in juvenile rats, but the sensitivity to NMDA blockers is strongly decreased in adult rats since the dose necessary to obtain a complete suppression of the windup is 10-fold higher than in juvenile rats.

In juvenile rats, windup of the nociceptive flexion reflex is always present if we suppress both one excitatory and one inhibitory synaptic component [11]. We tested for this possibility in adult rats. We first applied an inhibitory dose of 100 μg AP5 to suppress the windup (Figure 2D,E,G), we further applied both AP5 and strychnine, and the windup was recovered (Figure 2D,F,G). Thus, we demonstrated in adult rats that windup of the nociceptive flexion reflex depends on a synaptic balance between the excitatory and inhibitory components.

### 2.3. Windup of the Flexion Reflex Is Sensitive to IL Blockers in Adult Rats

**Figure 3:**
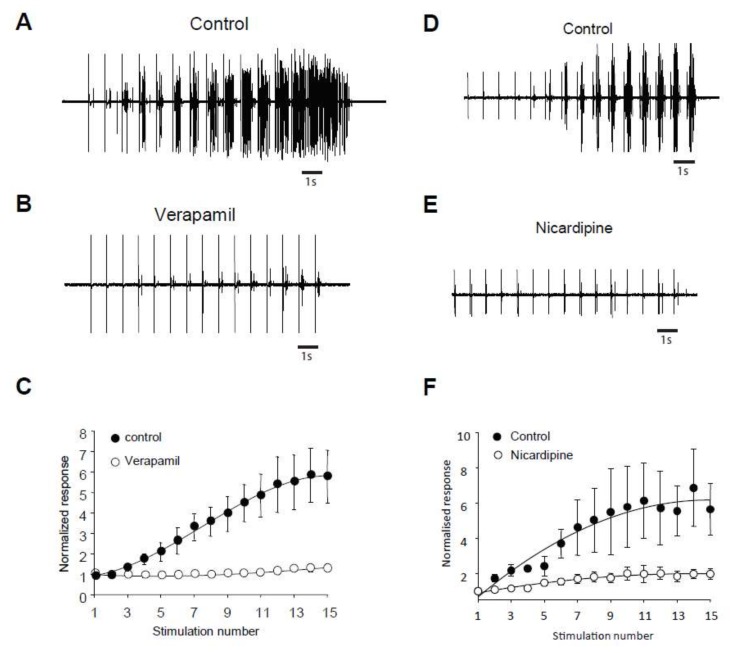
Flexion reflex windup depends on LTCs in adult rats. (**A**,**B**) EMG in control and after application of 100 µg of Verapamil. (**C**) Normalized response showing a significant decrease of flexion reflex windup (windup coefficient: 31 ± 9.8 in control vs. 5.4 ± 4 in verapamil, *n* = 5, *p* < 0.05, paired t-test). (**D**,**E**) EMG in control and after application of 100 µg of Nicardipine. (**F**) Normalized response showing a significant decrease of flexion reflex windup (windup coefficient: 62.5 ± 12 in control vs. 11 ± 3.3 in nicardipine, *n* = 5, *p* < 0.05, paired t-test).

**Figure 4:**
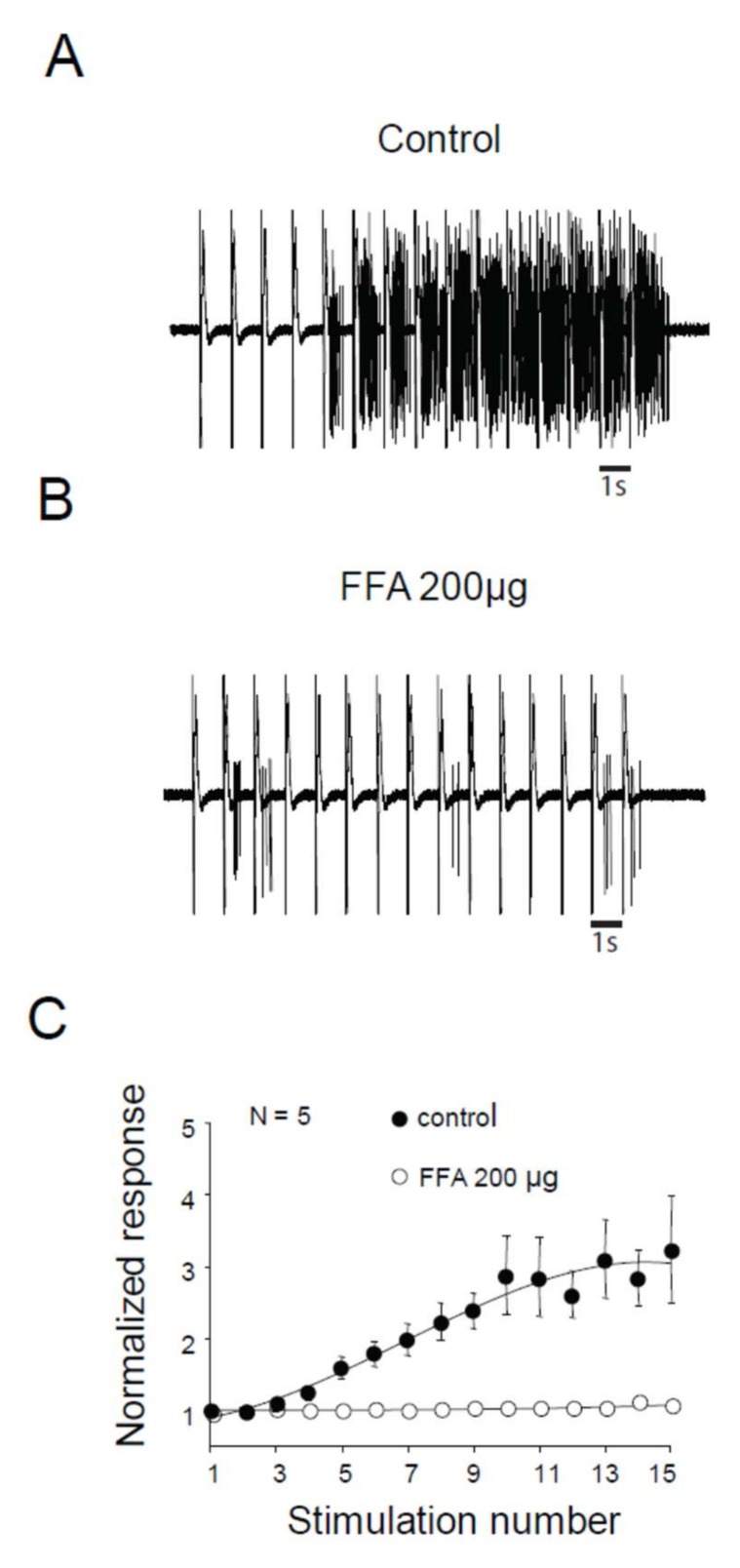
Flexion reflex in adult rats is sensitive to CAN currents. (**A**) and (**B**) EMG in control or after application of 200 µg of FFA. (**C**) Normalized response showing a significant decrease of flexion reflex windup with 200 µg of FFA (windup coefficient: 16.9 ± 4.7 in control vs. 0.16 ± 0.1 in FFA, *N* = 5, *p* < 0.05, paired *t*-test).

We assessed the role of ILs in windup of the flexion reflex, using two different families of blockers [14]. First, we applied 100 μg of verapamil, a blocker of the phenylalkylamine family. Verapamil blocked the windup of the flexion reflex (Figure 3A–C). The effect was dose-dependent with an EC50 of 26 μg. Because of the lack of specificity of phenylalkylamine, we used dihydropyridine to confirm the involvement of LTCs. Intrathecal application of 50 μg of nicardipine also suppressed the windup of the flexion reflex (Figure 3D–F). Again, the effect was dose-dependent with an EC50 of 16.4 μg. Finally, we assessed for the involvement of CAN in windup of the flexion reflex. Intrathecal Application of 200 μg of flufenamic acid (FFA) completely suppressed windup of the flexion reflex (Figure 4A,B). The effect of FFA was dose-dependent with an EC50 of 13.6 μg. These results demonstrate in adult rats that LTCs and CAN are two important elements in the expression of windup of the nociceptive flexion reflex.

### 2.4. Synaptic Component of Windup in DHNs of Adult Rats

**Figure 5:**
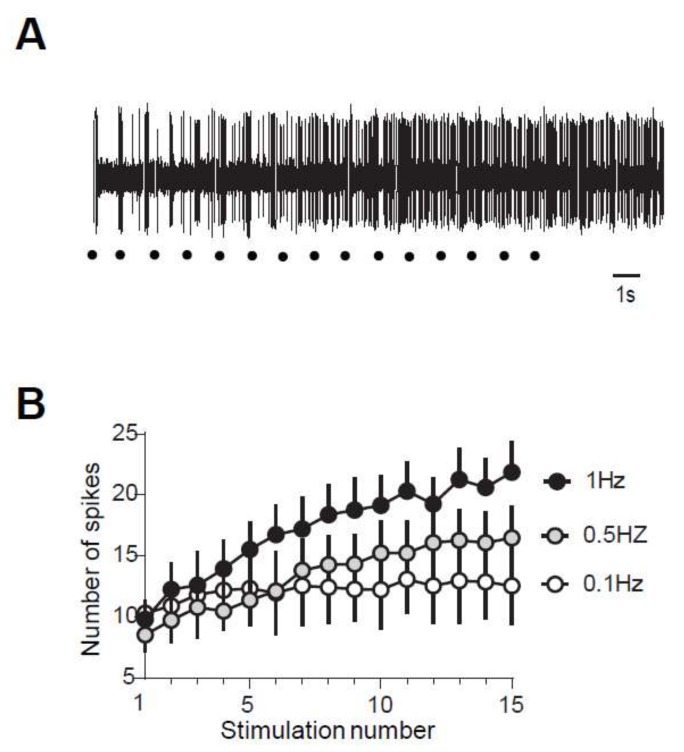
(**A**) Repetitive stimulations of the paw at three times the threshold for C-fiber induce a progressive increase in DHNs discharge showing up in a windup. (**B**) Windup of DHNs is frequency-dependent. Windup coefficient was 130 ± 15.7 at 1 Hz, 86.7 ± 24 at 0.5 Hz, and 29 ± 7 at 0.1 Hz, *n* = 7, 1 Hz vs 0.1 Hz, *p* < 0.05, Dunn’s multiple comparison test).

The nociceptive flexion reflex is the output of a reflex network that comprises two neuronal levels, i.e., dorsal and ventral horn. We then studied the properties of the windup of the discharge of DHNs and its sensitivity to a synaptic balance between excitation and inhibition. We first evaluated the characteristics of the windup in DHNs neurons in adult rats. We observed a progressive increase in neuronal discharge in response to repetitive stimulation of the hind paw at three-fold the threshold for C fibers in 53% (68/128) of the recorded neurons (Figure 5A). When present, windup was also frequency-dependent, with no windup at 0.1 Hz and an increased amplitude until 1 Hz stimulations (Figure 5B).

**Figure 6:**
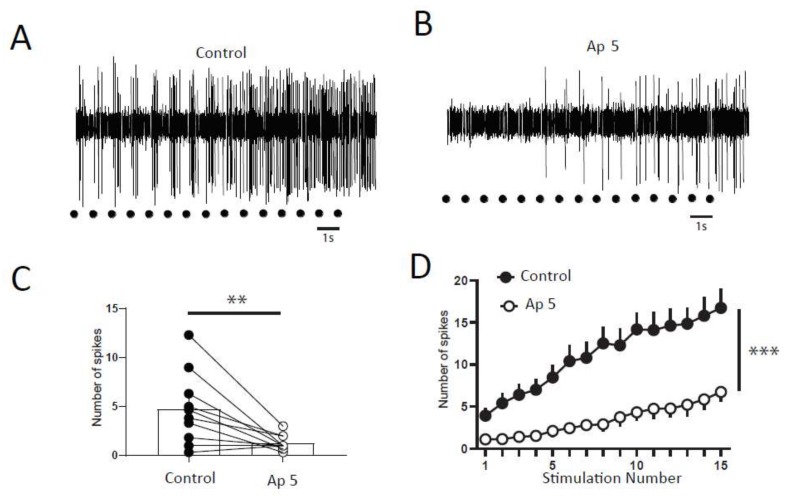
DHNs windup depends on NMDAr. (**A**,**B**) Extracellular recordings of DHN response to a series of electric shocks before and after 100 μg of AP5. (**C**) AP5 decreases DHNs excitability, since the response to the first nociceptive stimulation is significantly decreased (response to the first stimulation, 4.7 ± 1.2, spikes in control vs. 1.3 ± 0.25 spikes in AP5, *N* = 10, ** *p* < 0.01, paired *t*-test). (**D**) Amplitude of windup is significantly decreased (windup coefficient: 103 ± 15.7 in control vs. 31 ± 9.2 in AP5, *N* = 10, *** *p* < 0.001, paired *t*-test).

**Figure 7:**
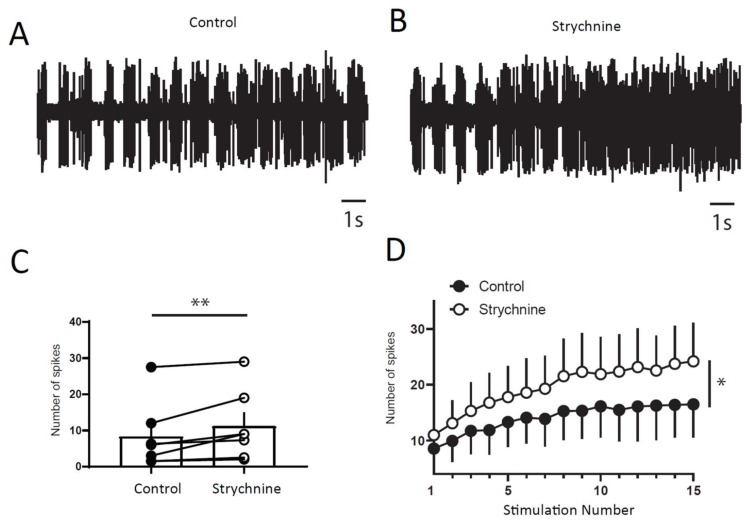
DHNs windup depends on glycinergic receptors. (**A**,**B**) Extracellular recordings of DHN response to a series of electric shocks before and after 170 μg of strychnine. (**C**) strychnine increases DHNs excitability, since the response to the first nociceptive stimulation is significantly increase (8.5 ± 3.5 spikes in control vs. 11 ± 3.6 spikes in Strychnine, *N* = 7, ** *p* < 0.01, paired *t*-test). (**D**) Amplitude of windup is significantly increased (windup coefficient: 83 ± 28.6 in control vs. 130 ± 43.3 in Strychnine, *N* = 7, * *p* < 0.05, paired *t*-test).

**Figure 8:**
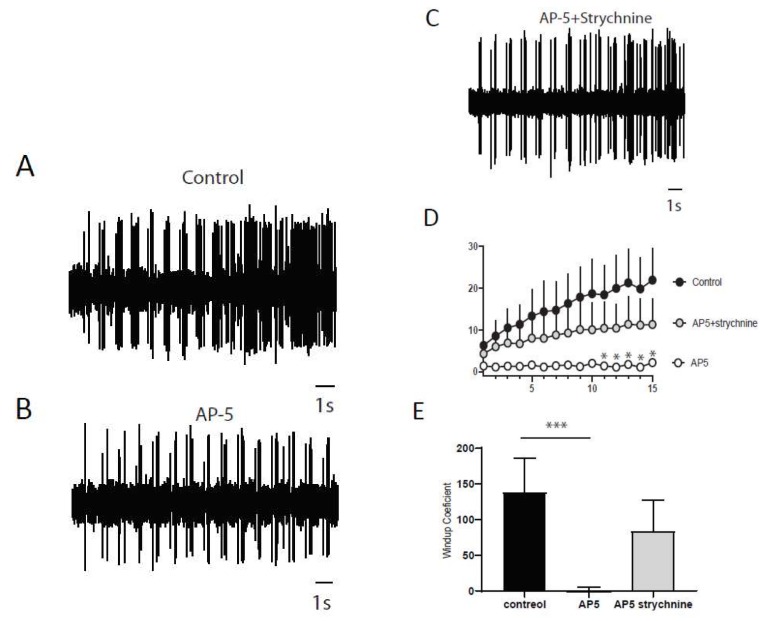
Dynamic synaptic balance control DHNs windup. (**A**) Extracellular recordings of DHN in control showing a windup. (**B**) After 100 μg application of AP5, windup is decreased. (**C**) Subsequent co-application of AP5 and strychnine restored a windup. (**D**) Windup curve showing difference between control and AP5 * *p*< 0.05 for stim n°11-15 and recovery with AP5 and Strychnine. (**E**) Windup coefficient is significantly decreased after AP5 and partially restored after AP5 and strychnine (windup coefficient 139.0 ± 47.07 in control; 0.2500 ± 5.155 in AP5; 84.33 ± 42.77 in AP5 + Strychnine, *N* = 8, *p*-control vs. AP5 < 0.001 (***), *p*-control vs. AP5 + Strychnine > 0.05, Dunn′s post hoc test).

Next, we wanted to determine if a balance between excitation and inhibition modulate windup of DHNs. We first confirmed that windup is influenced by NMDA receptor blockers (Figure 6). We showed that intrathecal application of 100 μg of AP5 significantly decreases DHNs excitability (Figure 6C) and Windup amplitude (Figure 6A,B,D). As already shown previously for the flexion reflex, inhibition control the amplitude of windup. To study this phenomenon in DHNs, we used strychnine to block glycinergic receptors (Figure 7). Intrathecal application of 170 μg of strychnine increased the excitability of DHN (Figure 7C). Windup was also increased (Figure 7A,B,D). Therefore, windup of DHNs of adult rats is potentiated by synaptic excitations and decreased by synaptic inhibitions. In the next step, we wondered if we could restore a windup after a blockade of NMDAr by suppressing inhibitory influence (Figure 8). To that issue, we suppressed windup of DHNs by applying 100 μg of AP5, and then we applied both 100 μg of AP5 and 170 μg of Strychnine. The 100 μg of AP5 almost completely suppressed the windup (Figure 8B,D,E), and the subsequent application of AP5 and Strychnine restored a windup of DHNs (Figure 8C–E).

### 2.5. LTCs Component of Windup in DHN in Adult Rats

**Figure 9:**
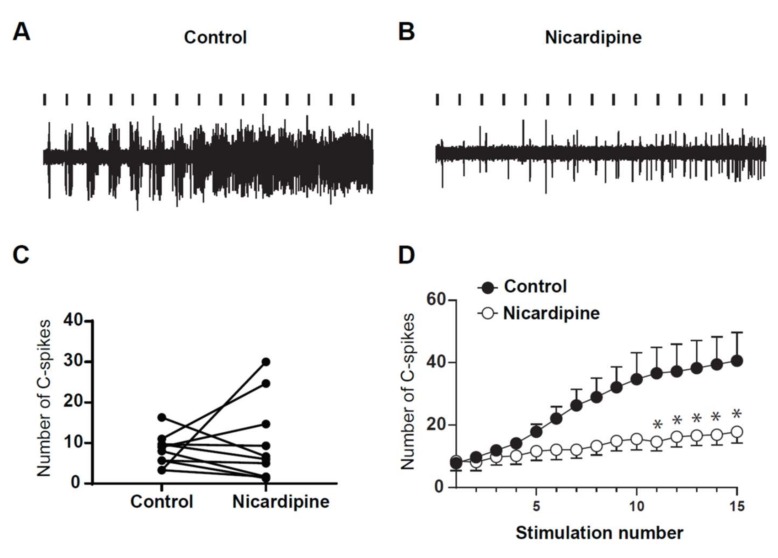
DHNs windup depends on LTCs. (**A**,**B**) Extracellular recordings of DHN response to a series of electric shocks before and after 100 μg of nicardipine. (**C**) nicardipine does not modify DHNs excitability, since the response to the first nociceptive stimulation remains unchanged (7.9 ± 1.34 spikes in control vs. 8.5 ± 3 spikes in nicardipine, *N* = 9, *p* = 0.84, Paired *t*-test). (**D**) Windup curve show decrease in amplitude under nicardipine (* *p* < 0.05 for stim n°11–15). Amplitude of windup is significantly decreased (windup coefficient: 280 ± 84 in control vs. 72 ± 29 in nicardipine, *N* = 9, *p* < 0.01, paired *t*-test).

Finally, we assessed for the role of LTCs in the expression of windup in DHNs of adult rats. To that issue, we used Nicardipine to block LTCs (Figure 9). Intrathecal application of 100 μg of nicardipine significantly decreased the amplitude of windup (Figure 9A,B,D). By contrast, neuronal excitability was not modified by nicardipine, since the DHN response to the first stimulation was not significantly modified. This result confirms previous results obtained in young rats and show that LTCs control the onset of windup, without altering normal neuronal communication.

Together, these results demonstrate that expression of windup by DHNs in adult rats depends on a synaptic balance between excitations and inhibitions and intrinsic properties.

## 3. Discussion

This study shows that the windup of a nociceptive flexion reflex depends on both synaptic and intrinsic components in adult rats in vivo. We confirmed the role of LTCs and CAN in this form of short-term sensitization to pain. We also showed the similar characteristics between the windup of nociceptive flexion reflex and the windup of DHNs, and we observed a global decrease in sensitivity to blockers in adult rats.

### 3.1. Adult and Juvenile Windup

These results demonstrate that the windup of the flexion reflex is not different between juvenile and adult rats. Indeed, as in juvenile rats, windup in adult depends on stimulation frequency and is sensitive to synaptic modulators and plateau potential blockers. Windup onset depends on a balance between synaptic inhibitory and excitatory inputs. However, we showed a general decrease in drug sensitivity in adult rats as compared to juvenile rats. These modifications in drug sensitivity have already been found for NMDA receptors blockers [16]. One may consider that differences between juveniles and adults could reflect a modification of neuronal phenotypes with altered expression in channels or receptors’ subunits. However, such a general effect for all tested substances is more likely due to an increased difficulty for any drugs to reach their targets. Indeed, the areas concerned are deep, and adult tissues have a complex extracellular matrix and are enriched in myelin.

### 3.2. Importance of LTCs in Windup

Windup is an activity-dependent short-term plasticity involved in pain processing. It represents a form of input/output amplification mechanism, which strengthens the contrast between background activity and relevant stimuli. Windup is known to depend on synaptic plasticity exerted through NMDAr and neurokinin [10,17,18]. In vitro recordings in juvenile rats revealed the role of intrinsic properties of DHNs in windup [12]. In brief, two ionic channels involved in the expression of plateau potentials are also necessary to trigger the windup of DHNs. These two channels are LTCs and CAN. LTCs are necessary in the early phase of plateau, whereas CAN promotes after-discharge [19]. However, the role of LTCs in short-term central sensitization to pain in adult rats is not fully demonstrated. For instance, in the formalin model of short-term sensitization, LTCs blockers have no effect [20], while LTCs blockers suppress DHNs windup [11]. Here, we show that, in adult rats, windup of a flexion reflex depends on LTCs since windup is blocked by blockers of LTCs belonging to two different families in a dose-dependent manner. We also confirm that windup of DHNs is also sensitive to LTCs blockers. Moreover, a recent study suggests that one specific type of LTCs is involved in windup in juvenile rats. Indeed, Two LTCs-forming channels are expressed in the dorsal horn of the spinal cord, Cav1.2 and Cav1.3. Blocking Cav1.3 expression with an antisense strategy suppresses the windup of the flexion reflex [13,14]. The participation of LTCs in pain depends on the type of pain. For instance, they are not involved in acute nociception, but they are clearly important for windup, central sensitization resulting from joint inflammation [21], and in long-term changes that accompany nerve injury [22], but not in sensitization induced by formalin injection [20].

We also show here that CAN is involved in the windup of the flexion reflex in adult rats. CAN promotes another important conductance of plateau potentials. CAN is triggered following LTCs activation and elicits a larger depolarization responsible for prolonged after-discharge [12,19]. The role of CAN in windup was demonstrated in DHNs’ neurons in slices from juvenile rats [19] or in anesthetized juvenile rats [11], and we show here their involvement in the windup of flexion reflex in adult rats.

### 3.3. Synaptic Component of Windup

The other major component of windup in vivo is synaptic. NMDAr and neurokinin receptors are activated during trains of stimuli, and this leads to the onset of windup. Here, we show that both windup of flexion reflex and windup of DHNs are completely suppressed by NMDAr blockers in vivo in adult rats, thus confirming classical results of the literature [10]. This blockade is also accompanied by a decrease in the baseline response, and DHNs’ excitability indicated an effect of NMDAr not only in sensitization but also in acute nociception [11]. We also show here that windup depends on inhibitory synaptic influences. Blocking glycine receptors increased the amplitude of windup of DHNs. This effect was accompanied by an increase in DHNs’ excitability. This effect is comparable to that previously observed in nociceptive flexion reflex [7,11]. This result confirms that inhibitory interneurons control the onset of central sensitization by exerting a tonic inhibitory tone. Finally, we show that windup in adult rats depends on a dynamic balance between excitation and inhibition. Indeed, when blocking NMDAr, we suppressed windup that could be restored by the subsequent blockade of the glycine receptor. This suggest that intrinsic properties of spinal neurons are a key element that elicits windup, as suggested in juvenile rats [11].

### 3.4. Neural Substrate of Windup

Two neuronal levels can elicit windup of their discharge within the flexion reflex circuit: motoneurons and DHNs [8,9,12,15,23]. We previously showed that, in control conditions, the windup of the flexion reflex was strictly correlated with DHNs windup [11]. In this condition, motor neurons seemed to follow amplification properties expressed by DHNs neurons. Here, we show that the windup of the nociceptive flexion reflex and the windup of DHNs share exactly the same properties, suggesting that the dorsal level is crucial to elicit reflex windup. Indeed, we show that both windup of flexion reflex and windup of DHNs are modulated by NMDAr and Glycine and controlled by LTCs and CAN. We cannot totally exclude the involvement of windup of motoneurons, but our results strongly suggest that the windup of the flexion reflex is mainly controlled at the dorsal-horn level.

### 3.5. Windup and Central Sensitization to Pain

Windup has long been studied in various models of pain. It has been defined as a model of short-term sensitization to pain, and it is considered to be a good model for the study of mechanisms leading to central sensitization to pain. Moreover, windup is still used to assess levels of pain in many models. For instance, there is evidence that shows the usefulness of windup as a cue of central sensitization in patients suffering from idiopathic pain [5,6]. However, windup is a short-term sensitization, with a timescale of a few seconds and cannot be responsible for long-term central modifications leading to neuropathic pain [24]. On the one hand, the timescale of windup is too short to trigger neuronal modifications appearing in long-term changes leading to an increase in spontaneous activity and an increased response to both innocuous and noxious stimuli. On the other hand, it seems that windup is not affected by nerve-lesions-induced neuropathy or slightly decreased [13,25].

Nevertheless, recent studies have shown that, in SNL rats, the number of neurons expressing plateau potentials increases dramatically as compared to naïve animals [26]. In conclusion, even if windup is not responsible for the deep changes appearing in central sensitization to pain, an increased capacity to generate windup in such pathology cannot be excluded in chronic pain syndromes, and controlling windup onset in the dorsal horn of the spinal cord is a potential way to limit exaggerated pain in chronic-pain syndrome.

## 4. Methods

### 4.1. Experimental Procedures

We used both juvenile (14 to 21 days old (55 g)) and adult Wistar rats in this study (250–350 g). All experimental procedures were approved by the local ethics (agreement number: APA3765, 10, November, 2017) committee, according to the International Association for the Study of Pain (IASP) ethical guidelines for experimental research, which we followed. At the end of each experiment, rats were killed with an overdose of anesthetics.

### 4.2. Drug Administration

Rats were anaesthetized with intraperitoneal urethane (1.1 mg/kg). A catheter (PE-10; Phymep, France) was inserted into the sub-arachnoid space, and the tip was pushed forward to the L4-L5 region. During recordings, drugs were injected using the catheter connected to a Hamilton syringe and flushed with 10 μL saline (dead volume of the catheter). Nicardipine and verapamil (Sigma-Aldrich, Saint-Louis, Missouri, USA) were freshly dissolved in saline. AP5 and strychnine (Sigma-Aldrich, Saint-Louis, Missouri, USA) came from 10 mM of stock solutions stored at −20 °C. Flufenamic acid (FFA) was freshly dissolved in dimethylsulfoxide (DMSO) (DMSO, Sigma-Aldrich, Saint-Louis, Missouri, USA) and then diluted with saline. For the dose–response curve, the lower dose was first intrathecally injected and responses measured. Each dose was separated by 30 min.

### 4.3. In Vivo Electromyographic Recordings

Recordings were made in the ipsilateral biceps femoris muscle with two Teflon-coated stainless-steel electrodes (Phymep, Paris, France; diameter 200 μm). Electrical stimuli (500 μs, single chocks, master 8 stimulator (A.M.P.I, Jerusalem, Israel) were delivered with electrodes placed under the paw skin in the region of the sural nerve. A maximum response was obtained for a stimulus intensity of 60 to 80 V, as determined with single shocks delivered at 30 s intervals, to avoid central sensitization. At this stimulus intensity, a slight muscle contraction was elicited without movement of the limb. Data were acquired by a CED 1401 interface and analyzed with Spike 2 software (CED, Cambridge, UK).

### 4.4. In Vivo Extracellular Recordings

After urethane anesthesia, rats were placed in a stereotaxic frame, to ensure stability during electrophysiological recordings. A laminectomy was performed on lumbar vertebrae L1–L3, for exposition of the L4–L5 segments of the spinal cord. Extracellular recordings of wide dynamic range dorsal horn neurons (DHNs) were made with borosilicate glass capillaries (2 MΩ, filled with 4% NaCl) (Harvard apparatus, Holliston, MA USA). Electrodes were positioned with a Microdrive (Unimécanique, France). The depth of the neurons from the dorsal surface of the spinal cord was monitored (recordings were performed between 500 and 1000 μm from the surface). The response to various natural stimuli (brush, pressure, and pinch) in the most responsive part of the receptive field of the neurons was characterized, to ensure the wide dynamic range properties of the recorded neurons. We recorded the neurons’ responses following transcutaneous electrical stimulation (see above) of the center of the receptive field. The criterion for the selection of a neuron was the presence of an Aβ-fiber-evoked response, followed by a C-fiber-evoked response. For analysis, we performed a post-stimulus histogram (PSTH) in 4 separate zones: an Aβ zone between 0 and 20 ms, an Aδ zone between 20 and 90 ms, a C zone between 90 and 300 ms, and a post-discharge zone (PD) between 300 ms and 1 s.

### 4.5. Windup Protocol

Sequences of 15 stimuli were delivered at 1 Hz, except in the experiment of Figure 1, in which the stimulation frequency was varied from 0.1 to 1 Hz, as indicated on the graph. For Flexion reflex, each stimulus was a single shock (500 μs) at ~80% of the intensity, eliciting a maximal response (see above). For extracellular recordings, single shock 3-fold the threshold for C-fibers.

### 4.6. Data Analysis and Statistics

The electromyographic signals were integrated by measuring the area under the curve after rectification of the signal. Windup plots (e.g., Figure 1A) were normalized to the first response of the series. For extracellular recordings, spikes in the C+PD part of the response were counted, and data were plotted with the Prism software (v 5.0, GraphPad Inc., San Diego, USA). All values are mean ± SEM, and N indicates the number of tested animals. A windup coefficient was calculated by dividing the sum of the 15 responses by 15 times the first recorded response. Comparison of windup plots were performed by using a two-way ANOVA. Windup coefficient comparison between two populations was performed, using the nonparametric Mann–Whitney test. Windup coefficient comparison between before and after drug applications were compared by using paired t-test, or a one-way ANOVA when 2 drugs were applied. A value of *p* < 0.05 was considered significant.
